# Associations between post-stroke motor and cognitive function: a cross-sectional study

**DOI:** 10.1186/s12877-021-02055-7

**Published:** 2021-02-05

**Authors:** Marte Stine Einstad, Ingvild Saltvedt, Stian Lydersen, Marie H. Ursin, Ragnhild Munthe-Kaas, Hege Ihle-Hansen, Anne-Brita Knapskog, Torunn Askim, Mona K. Beyer, Halvor Næss, Yngve M. Seljeseth, Hanne Ellekjær, Pernille Thingstad

**Affiliations:** 1 Department of Neuromedicine and Movement Science, Faculty of Medicine and Health Sciences, NTNU-Norwegian University of Science and Technology, Trondheim, Norway; 2 Department of Geriatric Medicine, St. Olavs Hospital, Trondheim University Hospital, Trondheim, Norway; 3 Department of Mental Health, Faculty of Medicine and Health Sciences, NTNU-Norwegian University of Science and Technology, Trondheim, Norway; 4 Department of Medicine, Bærum Hospital, Vestre Viken Hospital Trust, Drammen, Norway; 5 Department of Geriatric Medicine, Oslo University Hospital, Oslo, Norway; 6 Department of Radiology and Nuclear Medicine, Oslo University Hospital, Oslo, Norway; 7 Institute of Clinical Medicine, University of Oslo, Oslo, Norway; 8 Department of Neurology, Haukeland University Hospital, Bergen, Norway; 9 Centre for Age-Related Medicine, Stavanger University Hospital, Stavanger, Norway; 10 Institute of Clinical Medicine, University of Bergen, Bergen, Norway; 11 Medical Department, Ålesund Hospital, Møre and Romsdal Health Trust, Ålesund, Norway; 12 Stroke Unit, Department of Internal Medicine, St. Olavs Hospital, Trondheim University Hospital, Trondheim, Norway

**Keywords:** Stroke, Cognitive function, Motor function, Function

## Abstract

**Background:**

Motor and cognitive impairments are frequently observed following stroke, but are often managed as distinct entities, and there is little evidence regarding how they are related. The aim of this study was to describe the prevalence of concurrent motor and cognitive impairments 3 months after stroke and to examine how motor performance was associated with memory, executive function and global cognition.

**Methods:**

The Norwegian Cognitive Impairment After Stroke (Nor-COAST) study is a prospective multicentre cohort study including patients hospitalized with acute stroke between May 2015 and March 2017. The National Institutes of Health Stroke Scale (NIHSS) was used to measure stroke severity at admission. Level of disability was assessed by the Modified Rankin Scale (mRS). Motor and cognitive functions were assessed 3 months post-stroke using the Montreal Cognitive Assessment (MoCA), Trail Making Test Part B (TMT-B), 10-Word List Recall (10WLR), Short Physical Performance Battery (SPPB), dual-task cost (DTC) and grip strength (Jamar®). Cut-offs were set according to current recommendations. Associations were examined using linear regression with cognitive tests as dependent variables and motor domains as covariates, adjusted for age, sex, education and stroke severity.

**Results:**

Of 567 participants included, 242 (43%) were women, mean (SD) age was 72.2 (11.7) years, 416 (75%) had an NIHSS score ≤ 4 and 475 (84%) had an mRS score of ≤2. Prevalence of concurrent motor and cognitive impairment ranged from 9.5% for DTC and 10WLR to 22.9% for grip strength and TMT-B. SPPB was associated with MoCA (regression coefficient B = 0.465, 95%CI [0.352, 0.578]), TMT-B (B = -9.494, 95%CI [− 11.726, − 7.925]) and 10WLR (B = 0.132, 95%CI [0.054, 0.211]). Grip strength was associated with MoCA (B = 0.075, 95%CI [0.039, 0.112]), TMT-B (B = -1.972, 95%CI [− 2.672, − 1.272]) and 10WLR (B = 0.041, 95%CI [0.016, 0.066]). Higher DTC was associated with more time needed to complete TMT-B (B = 0.475, 95%CI [0.075, 0.875]) but not with MoCA or 10WLR.

**Conclusion:**

Three months after suffering mainly minor strokes, 30–40% of participants had motor or cognitive impairments, while 20% had concurrent impairments. Motor performance was associated with memory, executive function and global cognition. The identification of concurrent impairments could be relevant for preventing functional decline.

**Trial registration:**

ClinicalTrials.gov Identifier: NCT02650531.

## Background

Stroke is reported to be the third-most common cause of disability-adjusted life years (DALYs) worldwide [1]. Post-stroke motor and cognitive impairments are prevalent, and even though function improve during the first 3 months after stroke [2], approximately one fifth of stroke patients experience stroke-related disability 3 months post-stroke [3], highlighting the need of early detection and prevention of further deterioration of function. Motor deficits leading to impaired gait, balance and general reduction in physical function are seen in almost 50% of stroke cases 3 months after stroke [4]. The prevalence of mild and major cognitive impairments appears to vary from 14 to 29% and 11 to 42%, respectively, 3 months post-stroke, depending on the method used to define post-stroke neurocognitive disorder [5]. In a study assessing stroke patients 3-6 months post-stroke, Sachdev et al. reported a prevalence of mild cognitive impairment and dementia of 37 and 21%, respectively [6], underlining the importance of addressing cognitive impairments following stroke.

Assessments of motor and cognitive functions have established roles in the follow-up of stroke patients but have traditionally been studied, diagnosed and managed as distinct entities [7]. However, the stroke lesion itself, comorbid cerebrovascular disease and neurodegeneration may all cause both cognitive and motor impairments [8].

It is well-documented that older people in the general population with concurrent impairments in motor and cognitive functions are at increased risk of developing dementia as well as being at risk for higher rates of hospital admissions, falls, dependency and mortality [7, 9, 10]. In population-based studies, the simultaneous presence of gait disturbances and memory complaints, called motoric cognitive risk (MCR) syndrome, has also been shown to increase risk of developing dementia [10]. Additionally, in the stroke population, impaired balance and gait post-stroke are significant risk factors for cognitive impairment [11, 12]. However, previous studies are few in number, and there is a need for additional knowledge about the relationship between motor and cognitive impairments based on multicentre studies of stroke populations.

Previous research has clearly indicated an association between cognitive and motor performance among older people. Vascular pathology appears to be related to motor impairments and executive dysfunction, while impaired memory is a typical symptom of neurodegeneration, especially Alzheimer’s disease [13, 14]. Gait performance following stroke has been reported to be associated with global cognition, executive function and memory [11, 12, 15]. The inability to combine a cognitive task with a motor task like walking, assessed as dual-task cost, has been proposed as an early marker of dementia development [16]. However, studies of associations between motor function and different cognitive domains in older populations have found divergent results [10, 17–20], and there is little evidence in stroke populations. We hypothesized that motor performance would be more closely related to executive function than to memory in a stroke sample, reflecting vascular pathology. A recent consensus report recommended a minimum core battery for assessing the motor-cognitive interphase related to ageing and neurodegeneration in order to increase comparability across research studies, detect subtle or common reversible factors, and accelerate research on dementia, falls, and ageing-related disabilities [7]. This test battery included gait speed, dual-task cost of gait speed (DTC-speed), the Montreal Cognitive Assessment (MoCA) and the Trail Making Test Parts A & B (TMT-A & -B) [21–23].

The overall aim of this study was to describe concurrent impairment in cognition and motor performance and to explore how motor performance was related to global cognition, executive function and memory 3 months post-stroke.

## Methods

### The Nor-COAST study

The Norwegian Cognitive Impairment After Stroke (Nor-COAST) study is a prospective cohort study with participants recruited from five different hospitals in Norway between May 2015 and March 2017. The details of the study have been published elsewhere [24]. Inclusion criteria were as follows: a diagnosis of stroke according to the WHO criteria [25] or findings on magnetic resonance imaging (MRI) compatible with intracerebral haemorrhage or infarction; symptom onset within 1 week of admission; age > 18 years; the ability to communicate in Norwegian; and residing within the catchment area of the participating hospitals. Patients with expected survival < 3 months were excluded. The present study is a cross-sectional sub-study of the Nor-COAST study, utilizing data from the three-month follow-up. In addition to the inclusion and exclusion criteria for the Nor-COAST, the participants included in the present study had to have completed at least one cognitive and one motor assessment at three-month follow-up.

### Assessments

#### Participant characteristics

The National Institutes of Health Stroke Scale (NIHSS) [26] score at admission was used to measure stroke severity; possible scores range 0–42, with a higher score indicating a more severe stroke. Mild stroke was defined as NIHSS score 0–4 points, for moderate stroke 5–12 points, for moderate to severe stroke 16–20 points and for severe stroke > 20 points [4]. At three-moth follow-up, instrumental activities of daily living (IADL) were assessed with the Nottingham Extended Activities of Daily Living (NEADL), a 0–66-point scale where a higher score indicates greater independence [27], and the inability to walk 200 m was used as an indicator of physical frailty. Both assessments were based on information collected through interview of patient or caregiver. Functional dependency was measured with the Modified Rankin Scale (mRS) [28], which comprises six levels scored as 0–5 where 0 indicates no disability and 5 indicates severe disability; 6 is used to indicate death. Prestroke score on mRS was recorded at baseline though interview of caregivers, while status at 3 months was assessed by interview of patient or caregiver at the hospital outpatient clinic. The Charlson Comorbidity Index [29] was used as a descriptive measure to quantify comorbidity among the participants and was based on participant information and medical records collected at baseline.

#### Motor performance tests

The Short Physical Performance Battery (SPPB), an assessment of mobility, consists of three tasks: gait speed, assessed by 4-m timed trials; balance, assessed by the ability to stand for 10 s with the feet in three different positions; and leg strength, measured by the time required for five sit-to-stand movements from a chair. Each task is scored on a 4-point scale, with a total score ranging 0–12 and higher scores indicating better function [30]. The cut-off for a score indicating impairment was set at < 10 points [31]. To assess dual-task cost, the participants were first asked to walk 10 m at their preferred gait speed, and then walk the same distance while counting backwards [21]. Dual-task cost was calculated using the formula *([single-task gait speed – dual-task gait speed]/single-task gait speed × 100 = dual-task cost)* [16]; a reduction of more than 20% was characterized as impairment [16]. Grip strength was measured with a Jamar Hydraulic Hand Dynamometer® (Lafayette Instrument Europe, Loughborough, UK), and the highest score of three attempts using the stronger hand was applied in the analyses. Scores < 21 kg were characterized as impairment for women and < 37 kg for men [32].

#### Cognitive assessments

Global cognition was assessed with the Montreal Cognitive Assessment (MoCA) with possible scores ranging from 0 to 30 and higher scores indicating better cognition [23]. A cut-off for impairment was set at < 24 points, based on previous recommendations [33]. To assess executive function, the Trail Making Test Part B (TMT-B) [22] was applied. Taking more than 167 s (one standard deviation [SD] below normative mean for the age group 75–77 years) [34] to complete the test was defined as executive dysfunction. The 10-Word List Recall (10WLR), part of the 10-Word List Learning and Recall from the Consortium to Establish a Registry for Alzheimer’s Disease (CERAD) battery was applied to assess memory; a score < 5 was defined as impairment, in line with age-adjusted normative data [35, 36].

### Data collection

Baseline characteristics were retrieved from participants, their caregivers and medical records. Stroke diagnosis and NIHSS scores were assessed by stroke physicians during hospital stay. At three-month follow-up, participants were evaluated at a hospital outpatient clinic. Motor and cognitive assessments were performed by healthcare personnel who were trained to perform these tests and according to a standardized manual.

### Statistics

Demographic and clinical characteristics were summarized using mean and SD for continuous variables and frequencies and percentages for categorical variables. For analyses of prevalence, the clinical variables were dichotomized based on predefined cut-offs and investigated with cross tabulations between each of the motor and cognitive tests. Associations were studied using linear regression with the continuous variables of the cognitive tests as dependent variables and the motor measures as independent variables. First, regression analyses were performed for each combination of motor and cognitive assessments. Second, we conducted analyses for each of the cognitive tests one at a time, including all three motor assessments, as independent variables. All regression analyses were adjusted for age, sex, education and baseline NIHSS score, which had been pre-defined as plausible confounders. Residuals were checked for normality by visual inspection of Q-Q plots. Statistical significance was defined as two-sided *p*-value < 0.05, and 95% confidence intervals (CI) are reported where relevant. Data were analyzed using IBM SPSS Statistics for Windows, version 25 (IBM Corp., Armonk, NY, USA).

## Results

### Baseline characteristics

**Fig. 1:**
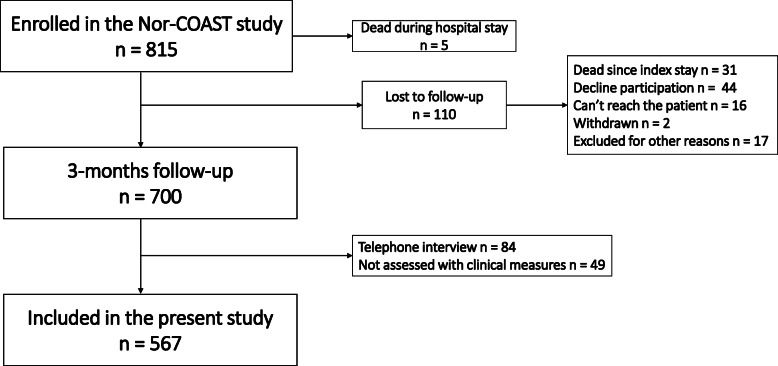
Flow chart on inclusion

Of 815 patients recruited to the Nor-COAST study, 700 were followed up at 3 months, and of these, 567 completed at least one motor and one cognitive assessment (Fig. 1). The mean (SD) age of the study participants was 72.2 (11.7) years; 242 (43%) were women; and 460 (82%) were diagnosed with ischemic stroke. Baseline mean (SD) NIHSS score was 3.7 (4.7), and 416 (75%) of the participants had NIHSS scores ≤4 points, indicating minor strokes (Table 1). The pre-stroke modified Rankin Scale (mRS) score was ≤2 in 533 (94%) of the participants, and after 3 months, 475 (84%) had an mRS score ≤ 2 (Table 1). As compared to study participants, those lost to follow-up were significantly older (mean [SD] age 78.5 [10.6], *p*-value < 0.001) and had suffered more severe strokes (mean [SD] NIHSS score 6.9 [7.8], *p*-value < 0.001), but there were no differences in sex.

**Table 1:** Baseline characteristics

	n		
Demographics			
Age, years, mean (SD)	567	72.2	(11.7)
Females, n (%)	567	242	(42.7%)
Living alone, n (%)	564	195	(34.4%)
Education > 9 years, n (%)	567	424	(74.8%)
Stroke classification			
Infarction, n (%)	558	460	(82.4)
Haemorrhage, n (%)	558	51	(9.0%)
Not classified, n (%)	558	47	(8.3%)
NIHSS score at admittance (0–42), mean (SD)	553	3.7	(4.7)
NIHSS score at admittance			
Mild stroke (0–4), n (%)	553	416	(75.2%)
Moderate stroke (5–15), n (%)	553	113	(20.4%)
Moderate to severe stroke (16–20), n (%)	553	20	(3.5%)
Severe stroke (> 20), n (%)	553	4	(0.7%)
Charlson Comorbidity Index, baseline (0–24), mean (SD)	567	3.9	(1.9)
Antiplatelet treatment at discharge, n (%)	567	388	(68.4%)
Anticoagulation treatment at discharge, n (%)	567	166	(29.3%)
mRS (0–6), pre-stroke, mean (SD)	565	0.7	(0.9)

*SD* standard deviation, *NIHSS* National Institutes of Health Stroke Scale, (0-42p), *mRS* Modified Rankin Scale (0-6p)

### Prevalence of motor and cognitive impairments

Scores for the motor and cognitive tests are presented in Table 2. SPPB scores < 10 points were found in 210 (37%) participants, indicating reduced mobility. DTC ≥ 20% was measured in 146 (29%) participants, indicating a clinically relevant reduction in dual task capacity, while grip strength was below cut-off in 97 (45%) of the women and 120 (39%) of the men, indicating a clinically relevant reduction of strength. On the cognitive tests, 185 (33%) scored below 24 points on the MoCA, indicating reduced global cognition; TMT-B > 167 s indicated impaired executive function in 194 (37%); and 10WLR score below five showed impairment in memory in 188 (39%) of the participants.

**Table 2:** Assessments at three-month follow-up

	n		
**Assessments of motor performance**			
SPPB (0–12), mean (SD)	563	9.4	(3.1)
Dual-task cost, (%), mean (SD)	500	12.3	(16.2)
Grip strength (kg), mean (SD)			
Men	305	40.1	(11.7)
Women	216	21.7	(6.9)
Gait speed 4 m, (m/s), mean (SD)	550	1.0	(0.3)
**Assessments of cognitive performance**			
MoCA (0–30), mean (SD)	562	23.8	(4.7)
TMT-B (0–300), mean (SD)	525	154.7	(83.9)
10-Word List Recall (0–10), mean (SD)	484	5.2	(2.7)
**Assessments of function**			
Able to walk 200 m, 3 months, n (%)	527	485	(92.0%)
mRS (0–6), 3 months, mean (SD)	565	2.0	(1.3)
Nottingham EADL (0–66), 3 months, mean (SD)	553	49.0	(13.0)

*SD* standard deviation, *SPPB* Short Physical Performance Battery (0-12p), *Dual-task cost* ([single-task gait speed – dual-task gait speed]/single-task gait speed × 100) (0–100%), *MoCA* Montreal Cognitive Assessment (0-30p), *TMT-B* Trail Making Test Part B (0–300 s), *10-Word List Recall* the recall part of the 10-Word List Learning and Recall from the CERAD (Consortium to Establish a Registry for Alzheimer’s Disease) Battery (0–10 words), *mRS* Modified Rankin Scale, *Nottingham EADL* Nottingham Extended Activities of Daily Living scale (0-66p)

**Fig. 2:**
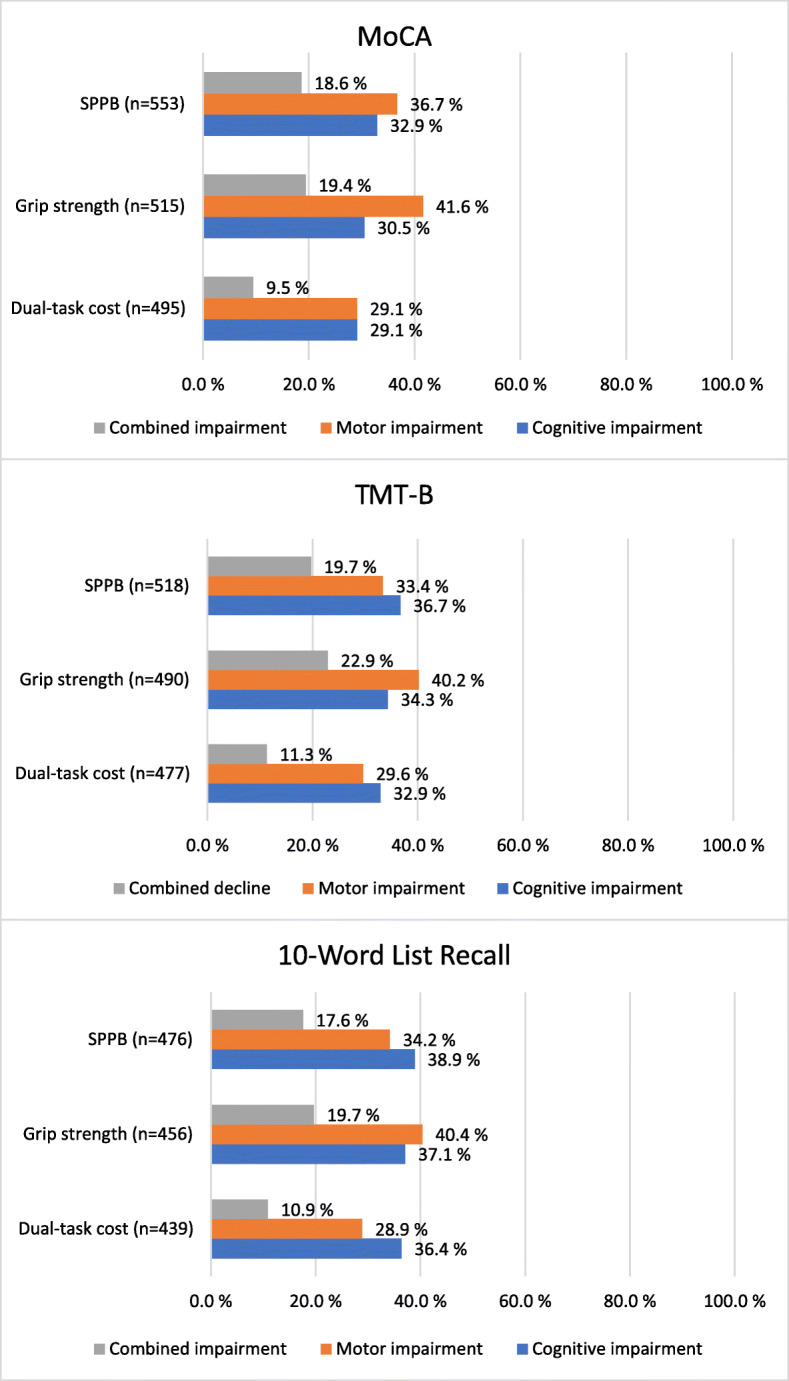
Prevalence of motor and cognitive impairments. *MoCA* Montreal Cognitive Assessment; *SPPB* Short Physical Performance battery; *Dual-task cost* ([single-task gait speed – dual-task gait speed]/single-task gait speed × 100); *TMT-B* Trail Making Test Part B. Impaired performance was defined as: MoCA <24p, TMT-B > 167 s, 10-Word List Recall < 5 words, SPPB < 10 p, Dual-task cost > 20% and grip strength < 21 kg (women) and < 37 kg (men)

Regarding the prevalence of concurrent impairment, dual-task cost in combination with each of the cognitive tests showed a prevalence of approximately 10% (range 9.5–11.3%). The other combinations of tests resulted in reduced scores for both the cognitive and motor tests in about one-fifth of the participants (range 18.6–22.9%) (Fig. 2). Of the participants who scored below 24 points on the MoCA, 103 (57%) scored below 10 points on the SPPB, and of those who took more than 167 s to complete the TMT-B or could not complete it, 102 (54%) scored below 10 points on the SPPB (Fig. 2).

### Associations between motor and cognitive function

As shown in Table 3, the regression analyses showed that both the SPPB score and grip strength were associated with scores on the MoCA (*p* < 0.001), TMT-B (*p* < 0.001) and 10WLR (*p* = 0.001). For example, the estimated regression coefficient B = 0.465 for SPPB with MoCA as dependent variable means that for two individuals with the same age, sex, education and stroke severity (NIHSS score at admission), and with one score difference in SPPB, the expected difference in MOCA is 0.465. The DTC was associated only with the score on the TMT-B (*p* = 0.005). These associations remained statistically significant in the adjusted model (Table 4).

**Table 3:** Regression analyses with one motor domain at a time as covariate^a^

	Regression coefficient			
	n	*B*	*p-value*	*95% CI*
**MoCA**				
SPPB	539	0.465	< 0.001	0.352, 0.578
Dual-task cost	485	−0.004	0.661	−0.023, 0.015
Grip strength	503	0.075	< 0.001	0.039, 0.112
**TMT-B**				
SPPB	507	−9.494	< 0.001	−11.726, −7.925
Dual-task cost	468	0.475	0.005	0.075, 0.875
Grip strength	479	−1.972	< 0.001	−2.672, − 1.272
**10-Word List Recall**				
SPPB	464	0.132	0.001	0.054, 0.211
Dual-task cost	430	0.000	0.951	−0.014, 0.015
Grip strength	446	0.041	0.001	0.016, 0.066

*MoCA* Montreal Cognitive Assessment, *SPPB* Short Physical Performance Battery, *Dual-task cost* ([single-task gait speed – dual-task gait speed]/single-task gait speed × 100), *TMT-B* Trail Making Test Part B

^a^adjusted for age, sex, education and stroke severity (NIHSS score at admission)

**Table 4:** Regression analyses with all motor domains as covariates in the same model^a^

	Regression coefficient		
	B	*p-value*	*95% CI*
**MoCA** (*n* = 463)			
SPPB	0.309	< 0.001	0.179, 0.438
Dual-task cost	−0.006	0.497	−0.025, 0.012
Grip strength	0.063	< 0.001	0.028, 0.097
**TMT-B (***n* = 448)			
SPPB	−8.588	< 0.001	−11.204, −5.972
Dual-task cost	0.499	0.009	0.124, 0.873
Grip strength	−1.613	< 0.001	−2.296, −0.930
**10-Word List Recall** (*n* = 417)			
SPPB	0.099	0.048	0.001, 0.198
Dual-task cost	−0.002	0.786	−0.017, 0.013
Grip strength	0.036	0.010	0.010, 0.061

*MoCA* Montreal Cognitive Assessment, *SPPB* Short Physical Performance Battery, *Dual-task cost* ([single-task gait speed – dual-task gait speed]/single-task gait speed × 100), *TMT-B* Trail Making Test Part B

^a^adjusted for age, sex, education and stroke severity (NIHSS score at admission)

## Discussion

In this cross-sectional study of survivors who had suffered mainly mild strokes, we found impairments in either cognitive function or motor function in about one-third of patients, while the prevalence of concurrent impairment ranged from 10 to 23%, depending on which combination of motor and cognitive domains were assessed. Impairments in mobility and grip strength were associated with impaired global cognition, executive dysfunction and impaired memory. Higher dual-task cost was associated only with executive dysfunction. The identification of concurrent impairments could be relevant for preventing functional decline and should encourage a holistic approach to this patient group.

The scores on post-stroke cognitive tests identifying impairment shown here are in line with the results of previous studies [37]. A trend of reduced mobility 3 months post-stroke, especially in patients having suffered from moderate stroke, has been described [38], and Vahlberg et al. [39] found SPPB scores in line with finding from the present study, which are lower than reported in the general population [40]. Motor function has been reported to be a significant predictor of cognitive decline after stroke [12]. However, associations between motor performance and cognitive domains have not, to the best of our knowledge, been examined previously in stroke samples. The dual-task cost has been recommended as a valid measure of the motor-cognitive interphase [41] and found to predict incident dementia in individuals with mild cognitive impairment without previous history of stroke [16]. Several studies of elderly persons with cognitive impairment have shown a strong association between the dual-task test and global cognition [10, 19], but this was not supported by a study of patients with Parkinson’s disease [42]. In the present study, dual-task cost was associated only with executive function.

In the present study, we have demonstrated that, although the patients suffered mainly mild strokes with low NIHSS scores, cognitive and motor impairments as well as concurrent impairments are prevalent, as the majority of patients with impaired MoCA scores also had impaired SPPB scores and vice versa. Concurrent motor and cognitive impairments have been shown to predict poor prognosis as, for example, an increased risk of developing dementia in stroke-free populations [43, 44], and there is reason to believe that these impairments can impact recovery and everyday life [45]. Therefore, we believe that, as part of the routine follow-up protocol after stroke, assessments of cognition and global motor functions should be performed to gain more information that may be relevant for prognosis and may indicate a need for continued rehabilitation even 3 months after a stroke [45]. Further cognitive function is very important for planning and performing rehabilitation and cognitive impairment, such as impaired memory or executive dysfunction, might change responsiveness to motor rehabilitation, which should be taken into consideration when developing targeted interventions in stroke populations [46]. We did not find support for motor performance being more closely associated with specific cognitive domains, and we suggest using global tests like MoCA and SPPB in order to assess cognitive and motor function.

Shared underlying pathologies might explain concurrent impairment in cognition and motor performance, for which there is increasing evidence [9]. Theoretically, the impairments can be caused directly by the stroke lesion or by structural and functional impairments that appear at a distance from the stroke lesion, also known as diaschisis [47]. Previous studies have shown that stroke survivors have small-vessel disease and neurodegeneration in addition to focal stroke lesions [48, 49], and small-vessel disease and neurodegenerative disease are both reported to be associated with impairments in gait and balance as well as cognition [8, 50–52]. Consequently, the observed impairments in motor and cognitive functions may be a symptom of both focal and disseminated brain pathology. The lack of findings of distinct associations could support a hypothesis of mixed pathology, but further research, including neuroimaging, is needed to achieve better insight.

The strengths of this work are the multicentre design, a relatively large sample size, and the comprehensive test battery that has been performed in line with consensus guidelines [7]. It is also a strength that the Nor-COAST participants are shown to be representative of the majority of the Norwegian stroke population that suffers from mild strokes [53]. Compared to the Norwegian Stroke Registry, the participants included in this sub-study were slightly younger (72 vs 73 years) with a larger proportion suffering from minor impairments (75% vs 69%) measured by baseline NIHSS scores [4]. Despite relatively wide inclusion criteria, there was a selection bias towards younger stroke patients with milder strokes. As a result, this sub-sample probably comprises those individuals most likely to benefit from interventions designed to prevent further functional decline and may be generalized to this part of the stroke population. The prevalence of impairments reported in this study is closely related to the choice of test battery and cut-off values, which are in line with current recommendations [7]. For MoCA, the cut-off for impairment was set at < 24 points, [33, 54] which should also detect patients with mild cognitive impairment in this population with elderly stroke patients. Because of the large scale of the study, we used a standardized protocol for dual-task cost with counting backwards. This could represent a methodological limitation in this heterogeneous sample, and individual adjustments such as applying more-complex cognitive tasks or motor performance tests could have resulted in other findings [55] but were deemed beyond the scope of this multicentre study. Lastly, the cross-sectional design of the study limits any conclusions in regard to causality.

## Conclusion

We found subtle cognitive and motor impairments and combinations of these to be relatively common among stroke survivors despite high premorbid functioning and minor strokes. Motor performance was associated with memory, executive function and global cognition. Our findings add knowledge about post-stroke motor and cognitive function and highlight the need for awareness of motor and cognitive impairments in stroke populations. Further research is needed in regard to the prognostic significance of our findings, as well as their associations to underlying pathology. Concurrent impairments should be recognized both in a short- and long-term perspective in order to identify and target those patients in need of prolonged rehabilitation to prevent further functional decline.

## Data Availability

The datasets generated and analysed during the current study are not publicly available due to Norwegian legal regulations. Requests to obtain anonymized study data can be addressed to the corresponding author.

## Abbreviations

10WLR: 10-Word List Recall
ADL: Activities of daily living
CERAD: Consortium to Establish a Registry for Alzheimer’s Disease
DTC: Dual-task cost
MoCA: Montreal Cognitive Assessment
MRI: Magnetic Resonance Imaging
mRS: Modified Rankin Scale
NIHSS: National Institutes of Health Stroke Scale
Nor-COAST: Norwegian Cognitive Impairment After Stroke
SPPB: Short Physical Performance Battery
TMT-B: Trail Making Test Part B
